# Catatonia associated with seronegative autoimmune encephalitis: a case report

**DOI:** 10.1097/MS9.0000000000003665

**Published:** 2025-08-07

**Authors:** Lily Rajbhandari, Prakriti Adhikari, Anil Nepali

**Affiliations:** a Department of Neurology, Kanti Children Hospital, Maharajgunj, Kathmandu, Nepal; b Department of Neurology, Kathmandu University School of Medical Sciences, Dhulikhel, Nepal

**Keywords:** case report, catatonia, immunomodulator, seronegative autoimmune encephalitis

## Abstract

**Introduction:**

Seronegative autoimmune encephalitis is a subgroup of encephalitis with suspected immunologic origin but with no identifiable pathogenic autoantibody in serum or cerebrospinal fluid (CSF).

**Case report:**

A 14-year-old girl presented with features suggestive of catatonia and altered mental status without any previous medical or psychiatric history and was subsequently diagnosed with seronegative autoimmune encephalitis. The patient showed notable improvement after immunomodulators (methylprednisolone) and lorazepam were initiated.

**Discussion:**

The absence of typical presentation along with absence of detectable serum or cerebrospinal fluid antibodies highlights the diagnostic and therapeutic challenges of autoimmune encephalitis (AE) in pediatric patients.

**Conclusion:**

The absence of detectable serum or cerebrospinal fluid antibodies does not exclude an autoimmune etiology when the clinical picture and supportive EEG findings are consistent with autoimmune encephalitis. Hence, autoimmune encephalitis should be suspected when presented with a rapid onset of psychological symptoms, and early and aggressive immunotherapy is crucial for favorable outcomes.

## Introduction

Autoimmune encephalitis is a spectrum of neuropsychiatric symptoms caused by acute inflammation of the brain secondary to the autoimmune processes^[1]^. The prevalence of autoimmune encephalitis is 13.7/100 000, making it the third leading cause of encephalitis^[2]^. The anti-NMDA receptor (NMDAR) antibody is the most common autoantibody detected in CSF or serum in the pediatric population^[3]^.

Seronegative autoimmune encephalitis refers to cases where traditional autoimmune markers are absent, making the diagnosis more challenging. The absence of detectable antibodies, despite the clinical features of autoimmune encephalitis(AE), poses significant diagnostic difficulties^[1]^. The diagnosis of autoimmune encephalitis is made even in the absence of autoantibodies in CSF or serum if the clinical probability for encephalitis is high because the diagnosis is primarily determined by clinical presentation, as antibody testing alone does not confirm it^[4]^.

We present the 14-year-old female with rapid-onset focal neurological deficit with catatonia with seronegative autoimmune encephalitis who responded well with immunotherapy after excluding possible psychiatric illness.

**HIGHLIGHTS**

- Seronegative AE presents with psychiatric symptoms, posing diagnostic challenges.
- Antibody absence hinders diagnosis; thorough testing is crucial.
- CSF, MRI, and EEG aid in identifying seronegative AE.
- Early immunotherapy improves outcomes in seronegative AE.
- Research on pathophysiology and antibodies can enhance diagnosis.

## Case report

A 14-year-old girl was brought by her parents to the outpatient department with the chief complaint of minimal verbal responsiveness for 7 days with immobility for 3 days. The symptoms were sudden in onset and progressive in nature. Initially, she had difficulty uttering words, struggled with initiating conversation, and had a lack of speech, followed by being verbally unresponsive to the majority of questions with an incomprehensible whisper. She showed minimal response to stimuli. She exhibited episodes of immobility with maintaining a rigid posture with flexion of bilateral arms, and attempts to move her limb resulted in mild resistance. Also, she had delusions of being pregnant. She was unable to perform basic activities of daily living such as brushing, toileting, and eating and needed assistance from her mother. She gradually became withdrawn, with reduced intake of food and also displayed insomnia and agitation. At times, she could not even recognize her mother. However, she had no history of fever, headache, vision changes, hallucinations, loss of consciousness, or abnormal body movement. Furthermore, she didn’t have a history of significant trauma, recent infections, or substance abuse. Her parents didn’t report any previous medical or psychiatric history. No clear trigger for the patient’s presentation was reported, including any recent stressors. There was no history of psychiatric illness in family members.

On examination, she was moderately built, well-groomed, and seemed confused and was not oriented to time, place, or person. However, her vital signs were stable. Systemic examination was unremarkable. On mental status examination, she appeared confused, with a decreased level of consciousness with a GCS of E4 V3 M5. Both her arms were flexed, with minimal movement. Her attention, concentration, and memory were also impaired. Her mother recalled her premorbid personality to be proactive, adventurous, and joyful. Her speech was sparse, and she responded to questions with single words and delayed answers with no echolalia. Affect was flat and non-reactive with hallucination with impaired insight and judgment. On neurological examination, mild rigidity in upper limbs, waxy flexibility, and poor concentration were noted with normal reflexes and no signs of meningism.

For these signs and symptoms, she was admitted to the psychiatric ward for further evaluation and management. The Bush-Francis Catatonia Rating Scale (BFCRS) was used to assess the presence and severity of catatonia, which showed mild catatonia with a total score of 5 out of 69.

**Figure 1.**
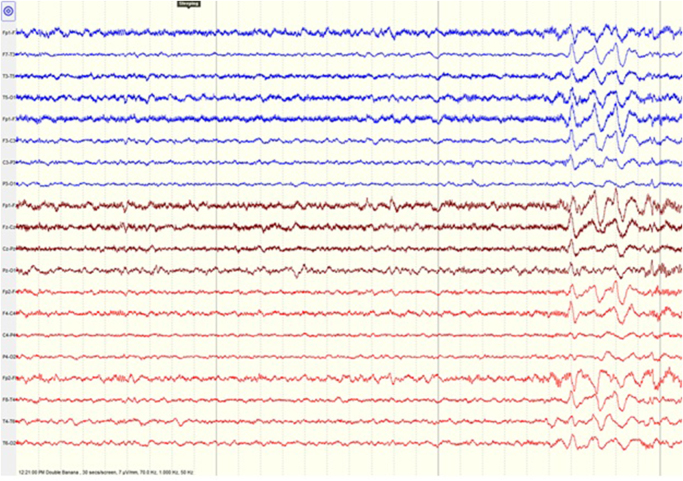
Electroencephalogram showing intermittent generalized slowing (delta activity) with delta brushing in the frontocentral region.

The patient’s initial laboratory tests, such as complete blood count, serum electrolytes, thyroid function, and liver function, were normal. A urine pregnancy test was negative. Brain and whole spine MRI showed no structural abnormality. The electroencephalography (EEG) showed intermittent generalized slowing (delta activity) with delta brush noted (Fig. 1), suggestive of encephalopathy, and advised to rule out anti-NMDAR encephalitis. Lumbar puncture was then performed for cerebrospinal fluid (CSF) analysis, which showed lymphocytic pleocytosis with glucose and protein within the normal range, suggesting inflammatory causes. However, CSF culture and polymerase chain reaction (PCR) for common viral pathogens were negative, ruling out infective causes. Also, serum and CSF tests included N-methyl-D-aspartate (NMDA) receptor antibodies, LGI1/CASPR2 antibodies, paraneoplastic antibodies, and anti-Hu antibodies that were negative. Serological testing was negative for HIV, influenza, adenovirus, rhinovirus, parainfluenza, enterovirus. Autoimmune investigations included antinuclear antibody, anti-neutrophil cytoplasmic antibody, and rheumatoid factor which were also negative. Erythrocyte sedimentation rate was 30 mm/hour. Chest X-ray and abdominal ultrasonography findings were done to rule out paraneoplastic causes, which were normal. Given the clinical presentation and the CSF findings, autoimmune encephalitis was suspected. However, tests for specific autoantibodies, including anti-NMDA-R and anti-LGI1 antibodies, were negative, confirming the seronegative nature of the disease. As patient presented with acute onset of altered mental status and catatonia, CSF analysis showed pleocytosis, negative well-characterized AE-associated antibodies and other differential diagnosis were excluded, she was diagnosed with probable antibody negative auto-immune encephalitis as per criteria for classification of pediatric autoimmune encephalitis.

Initially, 1 mg of lorazepam stat was given for catatonia and continued at 1 mg once a day. Once encephalitis was suspected, 1 g of methylprednisolone was given intravenously for 5 days, following which the patient showed notable improvement in her speech, which opened up gradually and became clear. Furthermore, she became oriented to time, place, and person gradually. Her final diagnosis was seronegative autoimmune encephalitis with catatonia. She was discharged on oral prednisolone on a tapering dose and oral olanzapine 7.5 mg once a day. Also, her vital signs were within normal limits at the time of discharge. And on follow-up after 14 days, the prednisolone dose was tapered and then stopped. In subsequent follow ups at 1 month and 3 months, patient had no signs of relapse or persistent cognitive and psychiatric deficits.

## Discussion

Catatonia in the pediatric population is rare, with high morbidity and mortality, and 20% of them have an underlying medical condition. Systemic lupus erythematosus and autoimmune encephalitis are the two common classes of autoimmune disorders associated with catatonia. Anti-NMDA-receptor (anti-NMDAR) encephalitis and pediatric autoimmune neuropsychiatric disorders associated with streptococcal infection are the most common autoimmune encephalitis causing catatonia^[5]^.

Autoimmune encephalitis is the immune-mediated acute inflammation of the brain. Patients usually present with various neuropsychiatric symptoms such as altered level of consciousness, confusion, disturbed sleep, movement disorders and seizures, behavioral changes, stereotypical behaviors, irritability, ataxia, chorea, dystonia, myoclonus, or tremor. In children younger than 13 years, new-onset psychosis is rare and considered a warning sign of a medical condition rather than a primary psychiatric condition^[6]^. In our case, the patient presented with catatonia and an altered level of consciousness.

The autoimmune encephalitis disease occurs as a result of a host’s immune system targeting self-antigens expressed within the central nervous system (CNS). The antibodies formed against neuronal cell-surface proteins, ion channels, or receptors are present in the CNS^[7]^. Autoimmune encephalitis can be further divided into three broad subtypes: (1) autoimmune limbic encephalitis, (2) acute disseminated encephalomyelitis, and (3) antibody-negative probable autoimmune encephalitis (ANPRA)^[8]^.

Whenever the patient presents with a spectrum of neuropsychiatric symptoms, acute in onset and progressive in nature, not explained by any other cause such as metabolic, vascular, toxic, or infectious, autoimmune encephalitis should be considered^[8]^. During the active phase of autoimmune encephalitis, abnormal findings are often noted in the EEG. EEG findings such as the “delta brush” pattern and extreme spindles suggest anti-NMDAR encephalitis, although it is of sensitivity. Autoimmune encephalitis is distinguished from primary psychiatric disorder with the help of the EEG, which shows epileptiform discharges and encephalopathic changes with diffuse or focal slowing waves^[8]^.

CSF analysis shows normal glucose and protein levels, with pleocytosis indicating the inflammatory condition. MRI of the brain may be either normal or abnormal, involving the temporal lobe, cortex, subcortex, and basal ganglia^[9]^. Seronegative autoimmune encephalitis, a variant of autoimmune encephalitis, is a diagnostic challenge in the pediatric population as there are no definitive criteria for diagnosis, despite the fact that certain criteria exist for the adult population with autoimmune encephalitis. Furthermore, antibody-positive status is present in only 44% of cases of autoimmune encephalitis^[3]^.

The classification of pediatric autoimmune encephalitis (AE) categories proposed by the subcommittee of the Autoimmune Encephalitis International Working Group is shown in Table 1^[6]^.

**Table 1:** Pediatric autoimmune encephalitis (AE) categories

1	Possible AE	Requires acute or subacute onset of neurological and/or psychiatric symptoms (≤3 months). Must have at least two clinical features of neurologic dysfunction (e.g., altered mental status, seizures, movement disorder, etc.). No requirement for paraclinical evidence (CSF, MRI, or biopsy findings), but their presence can support the diagnosis. Autoantibody testing is not necessary for classification. Other possible causes must be reasonably excluded before making this diagnosis.
2	Probable antibody-negative AE	Meets all criteria for possible AE (acute onset, ≥2 clinical features). Must have at least one piece of paraclinical evidence of neuroinflammation, such as: CSF abnormalities (elevated leukocytes >5 cells/mm^3^, oligoclonal bands). MRI showing encephalitis. Brain biopsy with inflammatory infiltrates. Autoantibody testing is negative or unavailable, meaning no well-characterized AE-associated antibodies are found. Other differential diagnoses must be excluded to confirm this classification.
3	Definite antibody-positive AE	Meets all criteria for possible AE (acute onset, ≥2 clinical features). Must have at least one paraclinical marker of neuroinflammation (CSF, MRI, or biopsy findings). Must have well-characterized AE-associated antibodies detected in serum or CSF. Exclusion of alternative diagnoses is mandatory.

In our case, the patient fulfilled the criteria for probable antibody-negative autoimmune encephalitis, as the psychiatric symptoms (catatonia and altered mental status) were present acutely over less than 3 months in the previously healthy female with a decreased level of consciousness with cognitive difficulties and EEG findings of generalized slowing (delta activity with delta brush waves fulfilling >2 criteria for clinical evidence of neurologic dysfunction). In paraclinical evidence of neuroinflammation, CSF analysis shows pleocytosis with the absence of well-characterized autoantibodies associated with AE in patient serum and CSF. All other possible causes of CNS inflammation were ruled out via investigation.

Thus, the diagnosis of autoimmune encephalitis was made despite the negative antibody results in CSF, as clinical suspicion was high. Antibody testing alone may not confirm the diagnosis because unidentified auto-antibodies may be involved in this case^[4]^.

Treatment of autoimmune encephalitis typically includes immunosuppressive therapies such as corticosteroids, intravenous immunoglobulin (IVIG), and plasmapheresis, as in this case. Early intervention has been associated with better clinical outcomes, though some patients may experience relapses or persistent cognitive deficits despite treatment. The patient in this case demonstrated a positive response to corticosteroid therapy with significant clinical improvement^[1]^. We treated our patient with intravenous corticosteroids for autoimmune encephalitis and oral lorazepam for catatonia.

In a case reported by Hah *et al*, an adult female with seronegative autoimmune encephalitis present with optic neuritis^[10]^. In another case report by Ismail *et al*, a young female with autoimmune encephalitis presents with clusters of epileptic seizures^[1]^. In contrast to these cases, our pediatric patient had catatonia and altered mental status. In a case series by Mansour *et al*, three patients developed seronegative autoimmune encephalitis shortly after COVID-19 vaccine^[11]^. However, our patient had no history of recent vaccination. In 14-year-old female with history of sexual abuse presented with catatonia, associated with anti-NMDR encephalitis resistant to benzodiazepams which responded to 1st and 2nd line immunotherapy as reported by Bogdan *et al*^[12]^. In contrast to this case our patient, catatonia was associated with probable antibody negative autoimmune encephalitis and was responsive to benzodiazepines.

## Parents’ perspective (with consent)

Initially, when our daughter couldn’t recognize us and became unresponsive, we were extremely distressed, fearing she might be suffering from a mental illness. In our society, there is a lot of social stigma surrounding mental health, which made us even more anxious about the health and future of our children. When we took her to Kanti Hospital, the doctors evaluated her and admitted her for further investigation. They told us she might have an infection in the brain, which confused and frightened us. However, the doctors reassured us and explained that with early treatment, there was a high chance of recovery. Fortunately, all the tests for infection came back negative, and our daughter began to gradually improve as treatment given to her as per the diagnosis made by. She was later discharged from the hospital. Now, she goes to school and plays normally with other children. We are very happy and grateful.

## Conclusion

This case illustrates the challenges in diagnosing and managing seronegative autoimmune encephalitis, a rare condition that can present with psychiatric symptoms, seizures, and cognitive decline. While the absence of detectable antibodies complicates diagnosis, a high level of suspicion and a comprehensive diagnostic approach – including CSF analysis, neuroimaging, and EEG – are critical for identifying this condition. Early treatment with immunosuppressive therapies such as corticosteroids and plasmapheresis can lead to significant clinical improvement. Future research into the pathophysiology of seronegative AE and the identification of novel autoantibodies is essential to improve diagnostic accuracy and treatment strategies.

## Data Availability

The data supporting this article’s findings are available from the corresponding author upon reasonable request.
